# Ion Channel Gradients in the Apical Tuft Region of CA1 Pyramidal Neurons

**DOI:** 10.1371/journal.pone.0046652

**Published:** 2012-10-03

**Authors:** Katie C. Bittner, Bertalan K. Andrasfalvy, Jeffrey C. Magee

**Affiliations:** HHMI Janelia Farm Research Campus, Ashburn, Virginia, United States of America; Institut National de la Santé et de la Recherche Médicale (INSERM U901), France

## Abstract

Dendritic ion channels play a critical role in shaping synaptic input and are fundamentally important for synaptic integration and plasticity. In the hippocampal region CA1, somato-dendritic gradients of AMPA receptors and the hyperpolarization-activated cation conductance (I_h_) counteract the effects of dendritic filtering on the amplitude, time-course, and temporal integration of distal Schaffer collateral (SC) synaptic inputs within stratum radiatum (SR). While ion channel gradients in CA1 distal apical trunk dendrites within SR have been well characterized, little is known about the patterns of ion channel expression in the distal apical tuft dendrites within stratum lacunosum moleculare (SLM) that receive distinct input from the entorhinal cortex via perforant path (PP) axons. Here, we measured local ion channels densities within these distal apical tuft dendrites to determine if the somato-dendritic gradients of I_h_ and AMPA receptors extend into distal tuft dendrites. We also determined the densities of voltage-gated sodium channels and NMDA receptors. We found that the densities of AMPA receptors, I_h,_ and voltage-gated sodium channels are similar in tuft dendrites in SLM when compared with distal apical dendrites in SR, while the ratio of NMDA receptors to AMPA receptors increases in tuft dendrites relative to distal apical dendrites within SR. These data indicate that the somato-dendritic gradients of I_h_ and AMPA receptors in apical dendrites do not extend into the distal tuft, and the relative densities of voltage-gated sodium channels and NMDA receptors are poised to support nonlinear integration of correlated SC and PP input.

## Introduction

Pyramidal neurons in hippocampal area CA1 receive spatial information from the entorhinal cortex both directly from layer III via perforant path (PP) axons and indirectly from layer II via Schaffer collateral (SC) axons. Each pathway makes synaptic contacts onto distinct regions of the CA1 dendritic arbor with SC axons synapsing onto apical and basal dendrites within stratum radiatum (SR) and stratum oriens (SO), respectively, while PP axons synapse onto more distal apical tuft dendrites in stratum lacunosum-moleculare (SLM). Without active mechanisms for signal amplification, the passive cable properties of dendrites filter distal synaptic events more than proximal events, resulting in location dependence of somatic voltage changes from widely distributed synapses. However, it is known that an increase in the number of AMPA-type glutamate receptors (AMPARs) and an increase in the density of distal hyperpolarization-activated cation current (I_h_) at distal synapses normalizes the amplitude, half width, and temporal summation of excitatory postsynaptic potentials (EPSPs) along the apical trunk of CA1 pyramidal neurons [1]–[6]. These compensatory mechanisms allow the SC pathway to operate as a single functional population of excitatory inputs to CA1 pyramidal neurons.

While somato-dendritic gradients of AMPARs and I_h_ current have been demonstrated for the SR segment of the apical trunk innervated by SC axons [7]–[10], it is unknown whether these gradients continue into the distal tuft dendrites. This is particularly relevant because the apical tuft receives functionally distinct input from the EC (PP) that appears to be integrated in a distinctly nonlinear mode through the generation of long duration dendritic plateau potentials that possess sodium (Na^+^), calcium (Ca^2+^) and NMDA dependent components [11]–[14]. To examine this issue we performed local dendritic measurements of the densities of synaptic AMPARs, the HCN channels that mediate I_h_ currents, voltage-gated Na^+^ channels, and NMDA-type glutamate receptors (NMDARs) both within distal SR and SLM. We found that the scaling of AMPAR and HCN channel density that occurs from proximal to distal SR does not continue into SLM. Furthermore, we observed that Na^+^ channel density is comparable between SR and SLM, but that the relative density of synaptic NMDARs with respect to AMPARs increases from SR to SLM.

## Methods

### Ethics Statement

All experiments were performed according to methods approved by the Janelia Farm Institutional Animal Care and Use Committee. This study was approved by Janelia Farm Institutional Animal Care and Use Committee (Protocol number 12–84). Animals were deeply anesthetized with 5% isoflurane before transcardial perfusion to ameliorate animal suffering.

Transverse hippocampal slices (300 µm–400 µm) were prepared using standard procedures [10] from 8–12 week old male Sprague-Dawley rats. Slices were cut using a Vibratome in an ice cold sucrose artificial cerebral spinal fluid (sACSF) containing (in mM) 234 sucrose, 28 NaHC0_3_, 2.5 KCl, 1.25 NaH_2_PO_4_, 7 MgCl_2_, 0.5 CaCl_2_, and 7 dextrose. Slices were incubated at 35°C in standard ACSF containing (in mM) 125 NaCl, 25 NaHC0_3_, 3 KCl, 1.25 NaH_2_PO_4_, 1 MgCl_2_, 1.3 CaCl_2_, and 25 dextrose bubbled with 95% O2–5% CO2 for 30 minutes and then stored at room temperature (∼1–6 h). Na pyruvate (3 mM) and ascorbic acid (1 mM) were added to the ACSF on the day of the experiment. Slices were visualized on a Zeiss Axioscope fitted with difference interference contrast optics and a 60× water immersion objective under infrared illumination (Na^+^, I_h_, and mEPSC data).

### Dendrite-attached Patch Methods

I_h_ and Na^+^ channel currents were measured using dendrite-attached patches from the apical trunk in distal SR (dSR, 250–350 µm from the soma), SLM (30–100 µm distal from the SR-SLM border) and/or proximal SR (pSR, 100–200 µm from the soma). Recordings were made using an Axopatch 200B amplifier (Molecular Devices, Sunnyvale, CA) and digitized at 20 kHz using an Instrutech ITC-18 with HEKA patchmaster acquisition software (Bellmore, NY). For I_h_ recordings, pipette solutions contained (in mM) 130 KCl, 10 HEPES, 2 CaCl_2_, 1 MgCl_2_, 20 TEACl, 5 4-AP, and 1 BaCl_2_. For Na^+^ channel recordings, pipette solutions contained (in mM) 130 NaCl, 3 KCl, 1.8 CaCl_2_, 1 MgCl_2_, 10 TEACl, 10 HEPES, 5 4-AP. Standard ACSF as described above was used for external solution at room temperature (22–25°C). Pipettes were pulled from thin-walled borosilicate glass (VWR, Radnor PA) using a Flaming/Brown micropipette puller P97 (Sutter Instruments, Novato CA) and had resistances of 10–18 MΩ. In some cases pipettes were coated with Sylgard (Dow Corning, Midland MI). Recordings were filtered at 2 kHz using an 8-pole Bessel filter. I_h_ recordings were additionally filtered at 500 Hz offline using a Gaussian filter. I_h_ currents were elicited with 1 s hyperpolarizing pulses to −135 mV from a holding potential of −35 mV at 0.2 Hz. Na^+^ currents were elicited with a range of voltage steps (−55, −45, −35, −25, −15, −5 mV) from a holding potential of −85 mV. Approximately 50–100 repetitions of the same voltage step were repeated to create ensemble averages at each potential. Current traces were leak and capacity corrected by subtracting the average of null traces (Na^+^) or a linearly scaled average trace (I_h_). Baseline currents were also adjusted manually if necessary. To estimate the number of Na^+^ channels in the patch, the maximum current was divided by the unitary current amplitude. Single channel current amplitude was estimated from patches where single channel events could be easily resolved.

### Whole Cell Current Clamp Recordings

#### I_h_ block experiments

Whole cell current clamp recordings were performed using a Dagan BVC-700 (Minneapolis, MN) in active “bridge” mode and digitized at 20 kHz using an Instrutech ITC-18 with HEKA patchmaster acquisition software. The external solution was the same as the standard ACSF described above and pipette solutions contained (in mM) 134 K-Gluconate, 6 KCl, 10 HEPES, 4 NaCl, 0.3 MgGTP, 4 MgATP, and 14 Tris-phosphocreatine. Recordings were made at 32–34°C. Pipettes had resistances of 5–12 MΩ. Recordings with series resistances greater than 50 MΩ were excluded from analysis. Voltages have not been corrected for liquid junction potentials. For I_h_ inhibition experiments, ZD7288 (10 µM) was added to the external solution and measurements were made after the membrane potential had stabilized and sag had disappeared (∼10 minutes). Apparent input resistance was estimated from the slope of the steady state voltage in response to a range of current injections (−200, −150, −50, 50) approximately 800–1000 ms after the start of the current injection. Local dendritic recordings were established in either distal SR or SLM.

#### NMDAR/AMPAR ratio

To measure NMDAR/AMPAR ratios, recordings were made from distal apical dendrites near the border of SR and SLM in longitudinally cut 400 µm thick slice [15] and EPSPs were elicited with stimulation of either PP axons or SC axons using a tungsten microelectrode (WPI, Sarasota FL) 800–1,700 µm away from the recording site. Stimulus intensity was set so that activation of isolated SC or PP inputs produced single EPSPs of amplitude less than 10 mV at the distal dendritic trunk (dSR) and apical tuft (SLM) to avoid spike generation. Whole cell current clamp recordings were performed using a Dagan BVC-700 (Minneapolis, MN) in active “bridge” mode and digitized at 20 kHz using a Prairie acquisition system (Prairie Technologies, Middleton, WI) on an Axio Examiner 1 (Carl Zeiss, Germany) microscope. The pipette solution was the same as described above. The external solution contained (in mM) 125 NaCl, 25 NaHC0_3_, 3 KCl, 1.25 NaH_2_PO_4_, 0.1 MgCl_2_, 2.6 CaCl_2_, 25 dextrose, 3 Na pyruvate, 1 ascorbic acid and 10 µM Glycine at 32–34°C. Slices were pretreated for at least 30 min before recording in low Mg containing ACSF. 10 µM SR95531, and 0.2 µM CGP were included in the external solution to block GABA(A)R- and GABA(B)R-mediated inhibition, respectively. ZD7288 (10 µM) was added to exclude any filtering affect on EPSP time-course by I_h_.

**Figure 1:**
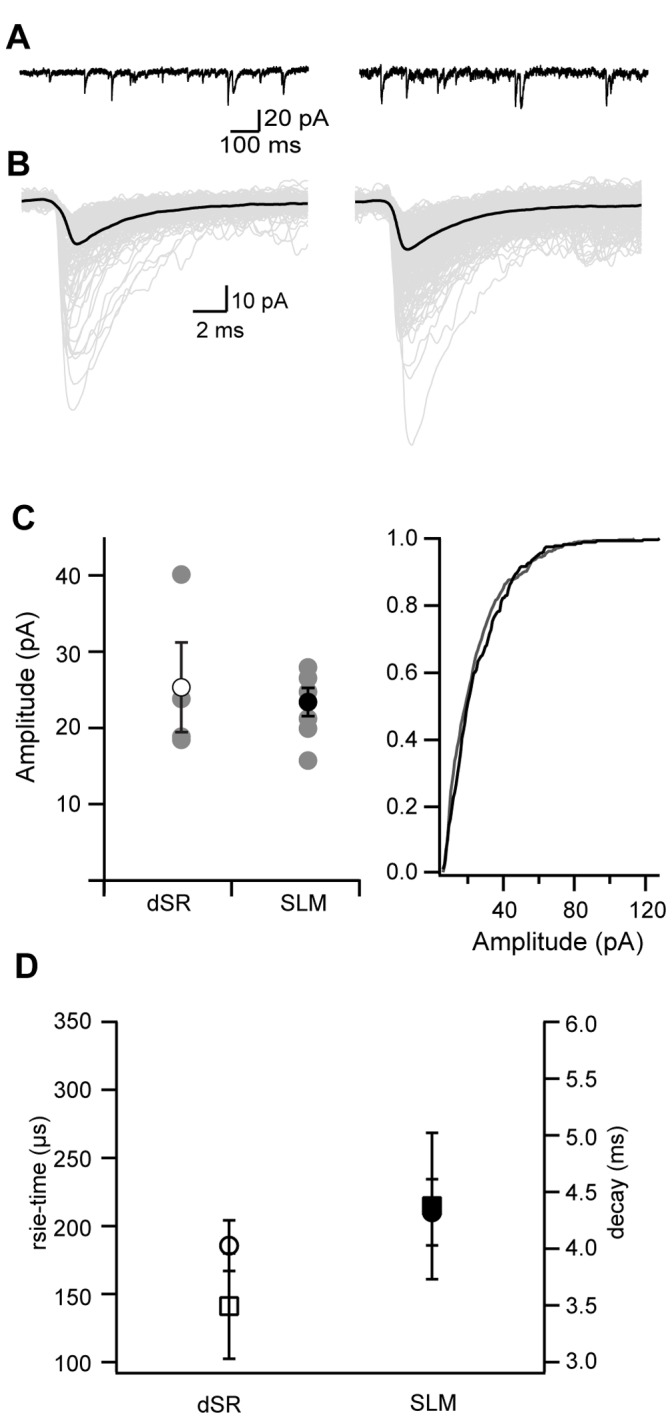
mEPSC amplitude is similar in distal SR and SLM. A. Representative current traces in response to a local pressure application of hyperosmotic sucrose solution recorded from dendrites within distal SR (left) or SLM (right). B. Representative average mEPSC events in black recorded in distal SR (left) and SLM (right) aligned by the start of an event. Individual EPSC events are shown in grey. C. Left, Average amplitude of mEPSCs recorded in distal SR (open symbol, n = 4) or SLM (closed symbol, n = 7) from all dendrites (black) and individual dendrites (grey). Right, cumulative probability distribution of mEPSC amplitude recorded in dSR (grey, n = 4) or SLM (black, n = 7). D. Average rise time (circle) and decay time (square) of mEPSCs from distal SR (open symbols, n = 4) and SLM (closed symbols, n = 7). Data are shown as mean ± SEM.

### Whole Cell Voltage-clamp Recordings (mEPSCs)

Whole cell voltage-clamp recordings were performed using a Dagan BVC-700 (Minneapolis, MN) in active “bridge” mode and digitized at 20 kHz using an Instrutech ITC-18 with acquisition software written in Igor. The normal external solution contained (in mM): 125 NaCl, 2.5 KCl, 1.25 NaH_2_PO_4_, 25 NaHCO_3_, 2 CaCl_2_, 1 MgCl_2_, 25 dextrose, bubbled with 95% O_2_–5% CO_2_ at ∼33–35°C (pH 7.4). All neurons had resting potentials between −55 and −75 mV. Series resistances from dendritic whole cell recordings were between 10 and 40 MΩ. mEPSCs were recorded from dendrites in distal SR (dSR, 250–350 µm from the soma) and SLM (50–150 µm from the start of the perforant path axons). To record miniature synaptic glutamate currents patch pipettes were filled with an internal solution containing (in mM): 120 potassium (K) gluconate, 20 KCl, 0.5 EGTA, 4 NaCl, 0.3 CaCl_2_, 4 Mg_2_ATP, 0.3 Tris_2_GTP, 14 phosphocreatine and 10 HEPES (pH 7.25). Unitary synaptic events were evoked by pressure ejection of a hyperosmotic external solution (with the addition of 300 mM sucrose), containing tetrodotoxin (TTX, 0.5 µM) and HEPES (10 mM) replaced the NaHCO_3_ (∼700 mOsm). AMPAR currents were isolated by the presence of external (+)-bicuculline (20 µM), and 0.5 µM CGP. Currents were recorded at −70 mV.

**Figure 2:**
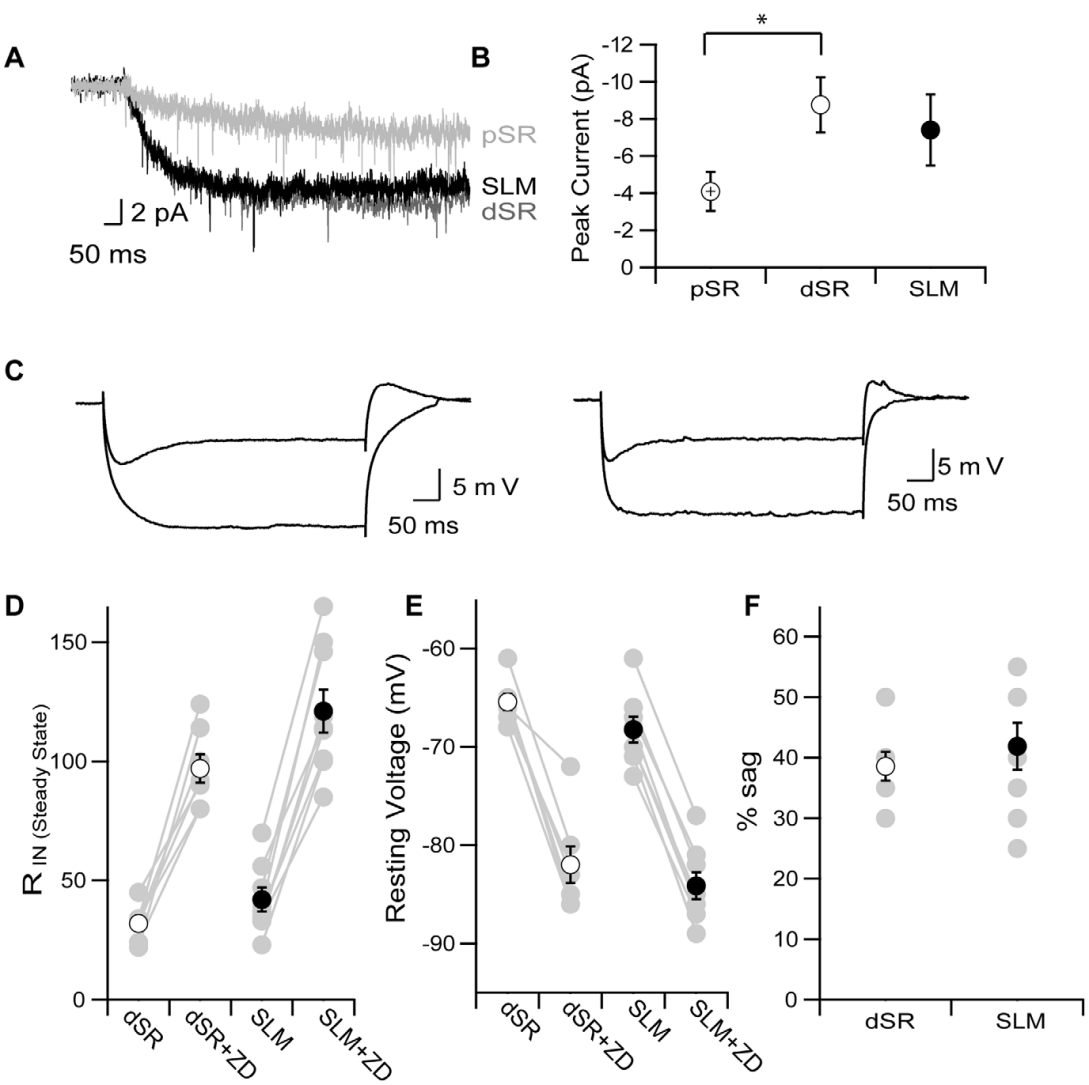
Distance dependent scaling of HCN channels in SR does not extend into SLM. A. Representative current traces from dendrite-attached patches in proximal SR (100–200 µm from the soma, light grey), distal SR (250–350 µm from the soma, dark grey) or SLM (30–100 µm distal from the SR-SLM border, black) in response to a step depolarization to −135 mV from a holding potential of −45 mV. B. Average peak current measured 800–1000 ms after the voltage step from dendritic patches in pSR (cross circle, n = 20), dSR (open circle, n = 78) or SLM (closed circle, n = 31). *denotes significance (p<0.027, Mann Whitney) C. Representative whole cell current clamp voltage traces in response to a −150 pA current injection before and after application of ZD7288 from dendrites in distal SR (left) and SLM (right). Average resting potentials were ∼18 mV hyperpolarized in ZD7288, however traces are shown offset for comparison purposes. D. Apparent input resistance measured as described in methods for dendritic recordings in either dSR (open circles) or SLM (closed circles) before and after application of ZD7288 (dSR n = 7, SLM n = 8). Averages are shown in black and individual are in grey. E. Resting membrane potential for dendritic recordings from either dSR (open circles) or SLM (closed circles) before and after application of ZD7288. Paired individual measurements are shown in grey and averages in black. F. Percent sag recorded in dendrites within dSR (open circles) or SLM (closed circles). Data are shown as mean ± SEM.

All data were analyzed using sigTOOL [16] and locally written programs in Matlab and Igor. Statistical analysis was done in GraphPad Prism and Matlab.

## Results

To determine whether the scaling of synaptic AMPAR density previously measured within the distal SR apical dendritic trunk continues into distal tuft dendrites of CA1 pyramidal neurons, we measured the amplitudes of miniature EPSCs (mEPSCs) using whole cell voltage-clamp of distal apical dendrites within SR (dSR) (250–350 µm from the soma) or tuft dendrites within SLM (50–150 µm from the start of the perforant path axons) in response to a local pressure application of hyperosmotic sucrose solution. Extrapolating from the increase in mEPSC amplitude observed in the apical trunk in SR we would expect an approximately two-fold increase in mEPSC amplitude between the dSR and SLM locations [3]. mEPSC amplitude was highly variable within both regions but on average was not different between the two regions (Figure 1, dSR 25.3±5.1, SLM 23.4±1.7 pA, ns Mann Whitney). To avoid inclusion of events from locations distal to the recording site, events with rise times greater than 400 µs were excluded. After exclusion of these events, average rise times and decay times were not different between the two regions (Figure 1D, rise time dSR: 186±16 µs, rise time SLM: 210±23 µs, decay time dSR: 3.5±0.4 ms, decay time SLM: 4.4±0.6 ms, ns Mann Whitney). These data suggest that the synaptic conductance does not continue to increase within the distal tuft.

**Figure 3:**
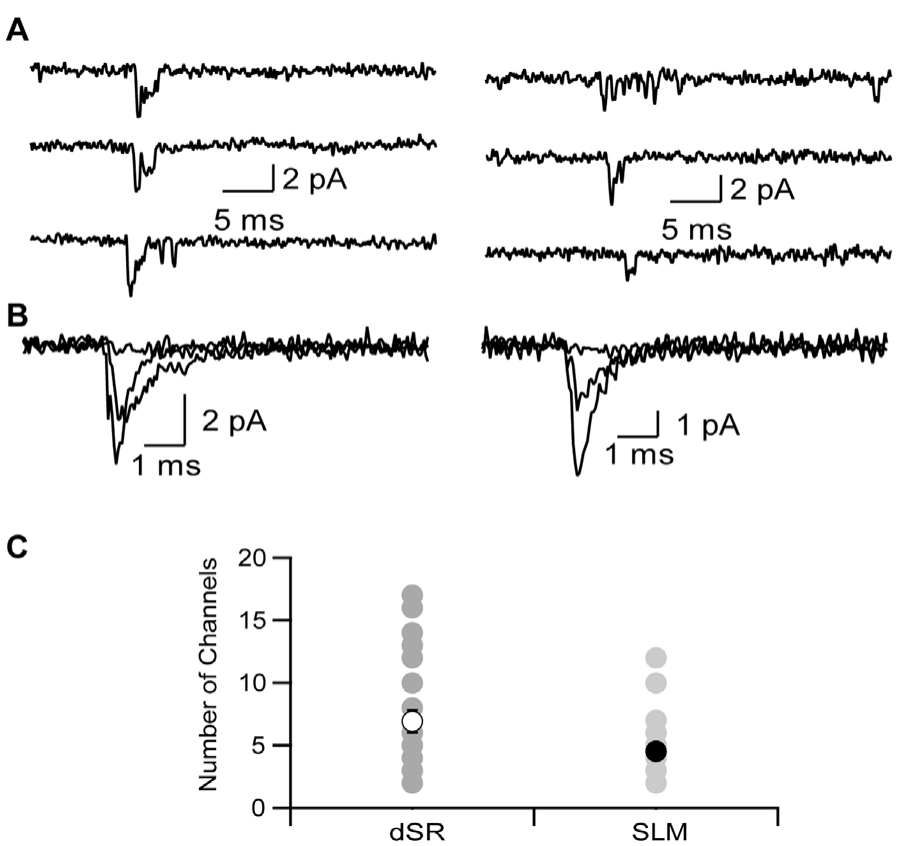
Na ^+^ **channel density is similar in SLM and distal SR.** A. Representative current traces from dendrite-attached patches in response to a voltage step from −85 mV to −25 mV in distal SR (left) and SLM (right). B. Representative ensemble average traces from the same dendrite as shown in A in response to voltage steps from −85 to −45, −25 and −5 mV. C. Average number of channels in dendrite-attached patches recorded in distal SR (open symbol) or SLM (closed symbol) calculated as described in methods. Data are shown as mean ± SEM in black and individual dendrites are shown in grey (dSR n = 26, SLM n = 19).

In addition to an increasing gradient of AMPARs in dendrites in SR, apical dendrites have been shown to have an increasing gradient of I_h_ current. To determine if I_h_ current density is higher in tuft dendrites within SLM compared to distal apical dendrites, we recorded currents in response to hyperpolarizing voltage steps from cell-attached patches on dendrites within SLM, distal SR, and proximal SR. The comparison between distal and proximal SR, where there is a known difference in current density, served as a positive control and ensured that the use of small aperture pipettes to patch the distal tuft dendrites did not limit our ability to detect potential differences. Figure 2A shows representative currents from cell-attached patches in proximal SR (100–200 µm from the soma), distal SR (250–350 µm from the soma), and SLM (30–100 µm distal from the SR-SLM border) in response to a voltage step to −135 mV from a holding potential of −35 mV. Based on the current density change recorded along the apical trunk, with recordings sites approximately 100 um apart, we would expect approximately a 1.5 fold increase in current amplitude between distal SR and SLM [10]. However, the average steady-state current measured after 800–1000 ms was not different between patches from dendrites in SLM compared to distal SR while the average current from patches in proximal SR were significantly smaller than the average current from distal SR patches (Figure 2B, pSR: 4±1 pA, dSR: 8.8±1.5 pA, SLM: 7.4±1.9 pA, p<0.027 Mann Whitney). These electrophysiological data are in contrast to fluorescence microscopy data which have reported increased labeling of the main Ih channel subunit HCN1 in the SLM compared to SR [8], [17]–[19].

**Figure 4:**
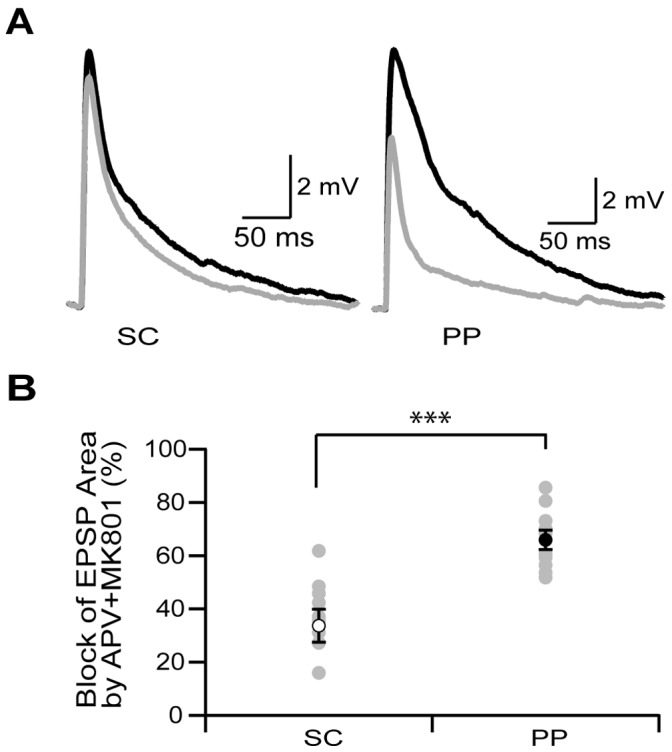
PP input to SLM recruits more NMDARs than SC input to SR. A. Evoked EPSPs in response to SC (left) or PP (right) stimulation in the absence (black) and presence (grey) of MK801 and APV. B. Percent of EPSP area blocked by MK801 and APV in response to SC (open symbol) or PP (closed symbol) stimulation (n = 10). ***denotes significance (p = 0.002, paired Wilcoxon). Average data are shown as mean ± SEM in black and individual patches are shown in grey.

While dendrite-attached patches are the most direct way to assess functional channel density, the use of this technique is limited because it requires very small pipettes, and the diameter of dendritic tuft branches decreases with length, becoming prohibitively small for patching beyond the first 100 µm into SLM. It would be difficult to observe a difference if the channels were clustered or if they are localized primarily in the smaller diameter dendrites. Therefore, we also used a secondary indirect measure to corroborate this result; we recorded from dendrites locally either in distal SR or SLM and used whole cell current clamp to estimate the effect of I_h_ blockade by ZD2788 on apparent input resistance and resting membrane potential. Based on the decrease in R_in_ from middle to distal SR we would expect a 50–75% decrease in R_in_ in the SLM location [10]. The percent decrease from peak to steady-state voltage (sag), apparent input resistances, and resting membrane potentials were similar in both regions before drug application (Figure 2C–2F, dSR sag: 39±2%, SLM sag: 42±4%, dSR R_in_: 32±3 MΩ, SLM R_in_: 42±5 MΩ, dSR V_m_: −65±1 mV, SLM V_m_: −68±1 mV, n = 7 dSR, n = 8 SLM, ns Unpaired T-test). Furthermore, changes in apparent input resistances and resting membrane potentials by application of ZD7288 were not different between the two regions (dSR R_in_ in ZD: 97±6 MΩ, SLM R_in_ in ZD: 121±9 MΩ, dSR V_m_ in ZD: −82±2 mV, SLM V_m_ in ZD: −84±1 mV, Fold change R_in_ dSR:3.2±0.4, Fold change R_in_ dSR:3.2±0.3 mV hyperpolarization in ZD dSR: 16.6±1.9, mV hyperpolarization in ZD SLM: 15.8±0.5 ns Wilcoxon Rank sum). These data are consistent with the previous conclusion that there is no difference in I_h_ current density between distal apical dendrite and the first 100 µm of distal tuft dendrites.

The above observations indicate that the ionic mechanisms shaping dendritic integration within the apical tuft are not simply a continuation of the mechanisms that exist in distal apical dendrites. Several previous studies have demonstrated that dendritic Na^+^ spikes and plateau potentials are important for propagation of tuft input to the soma [11]–[13], [20], suggesting that tuft dendrites contain channels to support these mechanisms. To determine the relative density of Na^+^ channels we measured single Na^+^ channel currents from dendrite-attached patches in both distal apical dendrites and tuft dendrites in response to depolarizing voltage steps (Figure 3). Patches from both regions typically contained greater than one channel, but single channel currents could still be resolved over a range of potentials. As expected for voltage-gated Na^+^ channels, increasing depolarization reduced latency to channel opening and increased the number of simultaneous channel openings. In patches that contained well-resolved single channel events and enough events to construct an amplitude histogram, the single channel current at −5 mV was estimated to be −1.2 pA (n = 6). The number of channels in each patch was estimated by observing the maximal number of simultaneous channel openings at a fully activating potential (−5 mV). There was no significant difference in the minimum number of channels in each patch from distal apical dendrites or tuft dendrites (Figure 3, dSR: 6.9±0.9, SLM: 4.5±0.6, p>0.07 Mann-Whitney).

In addition to Na^+^ spikes, Ca^2+^ plateau potentials have been shown to be important for transfer of information from the PP to the soma of CA1 pyramidal neurons [13]. NMDARs have been shown to be involved in controlling plateau initiation and duration. To determine the ratio of synaptic NMDARs to AMPARs, we recorded from distal trunk dendrites at the border of SR and SLM in current clamp mode and measured EPSPs in response to minimal stimulation of either SC or PP axons before and after application of APV and MK801 to block NMDARs. Experiments were performed in low (0.1 mM) magnesium (Mg) to alleviate voltage-dependent block of NMDARs at resting membrane voltage. Additionally EPSPs were measured in the presence of CGP 52646 and gabazine to block GABA receptors and ZD7288 to block I_h_. We observed that a larger fraction of mixed EPSP area was mediated by NMDARs at PP synapses than SC synapses (Figure 4, % block of EPSP area dSR: 34±6%, % block of EPSP area SLM: 66±4%, n = 10, p = 0.002 paired Wilcoxon).

## Discussion

Here we measured AMPAR mEPSCs, I_h_ currents, voltage-gated Na^+^ channel currents, and NMDAR/AMPAR ratios to determine the ionic mechanisms of synaptic integration within the distal apical tuft dendrites of CA1 pyramidal neurons. It has previously been shown that CA1 neurons compensate for distance-dependent attenuation of SC EPSP amplitude and time course through the expression of an increasing gradient of AMPARs and I_h_ current along the proximal to distal axis of the apical trunk within SR [1], [2], [7]–[10]. We found that this somatodendritic gradient does not extend into the apical tuft because mEPSC amplitude and time course, as well as I_h_ current density, is not different between the distal apical trunk and the tuft. The mEPSC data are consistent with EM immunogold localization data in that the data demonstrate that the gradient of AMPAR expression does not continue to increase into SLM, however there was a complicated relationship between synapse type and amount of AMPARs [7], [21], [22].

The I_h_ data, however, are in contrast to immunofluorescence and immunogold labeling studies which show an increasing expression of HCN1 channels along the proximal to distal axis that extends into the distal tuft [8], [17]–[19]. There are a number of possible reasons why our electrophysiological data are in contrast to previously reported immunofluorescence and EM immunogold localization data. Here we have measured functional channel density while imaging techniques measure total channel expression with limited ability to determine subcellular localization. Immunogold techniques are the best for determining subcellular localization, however the subcellular localization of HCN1 has only been carefully studied in a neighboring hippocampal region (subiculum) [8]. It is possible that increased fluorescence in SLM is due to channels that are either nonfunctional or not expressed within the membrane. However, it is also possible that there is in fact a higher density of HCN channels in tuft dendrites but they are localized in small diameter dendrites that were inaccessible to our recordings. Nonetheless, our data indicate that the I_h_ gradient present in the SR regions of the apical trunk does not extend into the dendritic regions within SLM and this strongly suggests that there is no further increase in I_h_ density within the apical tuft dendrites of CA1 pyramidal neurons.

The lack of conductance scaling and distance dependent increase in I_h_ amplitude in tuft dendrites suggest that this compartment uses a different integrative mechanism. One possible mechanism is the generation of dendritic spikes or plateau potentials and voltage-gated Na^+^ channels and NMDARs have both been shown to be important for these types of dendritic nonlinearities. Here we found that the Na^+^ channel density is similar in the tuft relative to the distal apical trunk of CA1 dendrites, extending previous electrophysiological data demonstrating constant Na^+^ channel density along the apical trunk in SR [23]. These data are in slight contrast to an EM immunolocalization study by Lőrincz and Nusser which has shown that the density of the voltage-gated Na^+^ channel subunit Na_V_ 1.6 decreases along the proximal to distal axis [24]. In the study by Lorincz and Nusser, they also stained for two additional isoforms that have been found in the central nervous system, Na_V_ 1.2 and Na_V_ 1.1, but immunofluorescence was not sensitive enough to detect any dendritic labeling of these proteins [24]. Without more sensitive antibodies to determine whether there are additional isoforms that account for the difference between the imaging data and electrophysiolgical data it is difficult to determine whether the functional channel density is constant throughout CA1 dendrites because of the expression of multiple isoforms or if there is some modification of Na_V_ 1.6 to account for the difference in expression and functional channel density.

In addition to Na^+^ channels, NMDARs have been shown to enhance dendritic excitability through their contribution to plateau potentials [13]. Here we found that the NMDAR/AMPAR ratio is higher at PP synapses compared to SC synapse. These data are in agreement with previous somatic whole cell voltage-clamp recordings that demonstrated a larger NMDAR/AMPAR ratio for PP stimulation over SC stimulation [25]. Here we recorded EPSPs locally from dendrites at the border between SLM and SR in low Mg to estimate the NMDAR/AMPAR ratio because NMDAR/AMPAR ratios measured using somatic whole cell voltage-clamp recordings are sensitive to large space clamp errors. These data along with the AMPAR mEPSC data suggest an increased synaptic current mediated by NMDARs. This is in contrast to immunogold localization data which found no change in NMDAR expression along the length of CA1 dendrites [7].

CA1 pyramidal cells are thought to perform an input comparison type of computation on two distinct input pathways, the PP and SC [11]–[13]. SC axons synapse throughout the more proximal regions of the apical and basal dendrites while PP axons synapse on the distal most apical tuft of CA1 dendrites. Several previous studies have shown that the impact of SC synapses is independent of their distance from the soma. This is advantageous to CA1 cells because it allows the impact of SC synaptic input to be determined by factors other than the dendritic location of the input. PP axons carry different spatial information and studies have suggested that synaptic integration of these inputs is different from SC inputs in that it is more nonlinear and involves the initiation of dendritic spikes and plateau potentials [11]–[13]. Here we found that the channel profiles of the apical tuft dendrites, particularly the presence of Na^+^ channels and an increased NMDA/AMPA ratio, support the generation of dendritic spikes and Ca^2+^-dependent plateaus. In summary it appears that the dendritic and synaptic mechanisms in place to normalize SC inputs do not extend to PP inputs in CA1 pyramidal neurons. Furthermore, the characterized ionic mechanisms support a unique form of dendritic integration in the apical tuft that enhances the interaction between correlated PP and SC input. These ionic mechanisms could also affect the rules that govern synaptic plasticity of PP inputs. Long term potentiation of PP inputs have been shown to be dependent on the generation of local voltage-gated Ca^2+^ channel and NMDAR dependent dendritic spikes rather than backpropagating action potentials [11]. An increase in functional NMDAR current could lower the threshold for LTP induction though increased Ca^2+^ entry.

In addition to Na^+^, I_H_, NMDAR, and AMPAR, voltage-gated Ca^2+^ and K^+^ channels heavily contribute to dendritic excitability and synaptic integration [23], [26]. These channels are likely to be particularly important in controlling the initiation and duration of Ca^2+^-dependent plateau potentials that are a prominent feature of the distal apical dendrite excitability profile. Future experiments examining the voltage-gated Ca^2+^ and K^+^ channel properties of the apical tuft should add to our understanding of plateau generation and the input comparison that they produce in hippocampal CA1 pyramidal neurons.
